# Different Chronic Disorders That Fall within the Term Juvenile Idiopathic Arthritis

**DOI:** 10.3390/life11050398

**Published:** 2021-04-27

**Authors:** Lucia M. Sur, Remus Gaga, Emanuela Duca, Genel Sur, Iulia Lupan, Daniel Sur, Gabriel Samasca, Cecilia Lazea, Calin Lazar

**Affiliations:** 1 Department Pediatrics I, University of Medicine and Pharmacology “Iuliu Hațieganu”, 3400 Cluj-Napoca, Romania; luciasur@umfcluj.ro (L.M.S.); cecilialazea@umfcluj.ro (C.L.); calinlazar@umfcluj.ro (C.L.); 2 Pediatrics I Department, Emergency Clinical Hospital for Children, 3400 Cluj-Napoca, Romania; 3 Pediatrics II Department, Emergency Clinical Hospital for Children, 3400 Cluj-Napoca, Romania; remusgaga@umfcluj.ro (R.G.); emma_floca@umfcluj.ro (E.D.); surgenel@umfcluj.ro (G.S.); 4 Molecular Biology Department, Babes Bolyai University, 3400 Cluj-Napoca, Romania; iulia.lupan@ubbcluj.ro; 5 The Oncology Institute “Prof. Dr. Ion Chiricuta”, 3400 Cluj-Napoca, Romania; dr.geni@umfcluj.ro

**Keywords:** juvenile idiopathic arthritis, diagnosis, subtype, subclassifications

## Abstract

Juvenile idiopathic arthritis (JIA) represents a significant challenge for pediatricians who intend to diagnose and treat this pathology. The classification criteria for JIA subtypes are rigid and often do not fully satisfy the possibilities of classification in the subtype. The objective of this study was to identify clearer criteria for classifying JIA subtypes. The 2019 expert committee meeting (PRINTO) shows the difficulties of this classification and proposes new research directions for the identification of disease subtypes. Four different chronic disorders are used to define JIA in a concise and easy to follow classification system. However, dates from the literature suggest that at least 10% of cases are still difficult to classify. Possibly in the future, different classifications of JIA based on pathophysiological and genetic criteria would be necessary.

## 1. Introduction

Juvenile idiopathic arthritis (JIA) is the most common chronic rheumatologic disease of childhood and is one of the most frequent chronic conditions in children. The prevalence of JIA during childhood is between 16 and 150 per 100,000 in Europe, respectively, and from 2 to 20 per 100,000 in North America [1,2].

The etiology is not fully understood; hence the term “idiopathic” has become increasingly used lately. Genetic research has found complex interactions between the genes involved in the pathophysiology of the disease, so clear distinctions between the various subtypes are difficult to make.

Due to these, JIA is often an exclusion diagnosis that can be easily missed by inexperienced pediatricians. The aim of this review is to present JIA as an entity defined by four different disorders based on the latest PRINTO classification. Additionally, we aim to show how even this new classifying system can have its inadequacies when trying to categorize JIA and could be further improved by pathophysiological and genetic criteria.

## 2. A history of Different Classification Systems

There have been attempts to classify JIA: first in 1977 with six subsets, and then in 1986 with three subsets. The current classification was proposed in 1997 by the International League of Associations for Rheumatology (ILAR). According to the consensus conference of the ILAR in 2001 at Edmonton, this classification has been revised to its final form, consisting of seven JIA categories: (a) oligoarthritis, (b) polyarthritis (rheumatoid factor positive), (c) polyarthritis (rheumatoid factor negative), (d) systemic arthritis, (e) psoriatic arthritis, (f) enthesitis-related arthritis and (g) undifferentiated arthritis [2,3] (Table 1).

1. Systemic JIA—first of all, it is characterized by the appearance of fever of unknown origin with the exclusion of auto-inflammatory, autoimmune, infectious and neoplastic diseases. The characteristics of fever consist of a fever (up to 39 degrees Celsius) once a day with a return to normal between the peaks. Fever should be accompanied by one or more of the following: (1) evanescent erythematous rash; (2) generalized lymph node enlargement; (3) hepatomegaly and/or splenomegaly; 4. serositis.

There were four excluded criteria that ILAR considered of no further use in the diagnosis of JIA:

a. Psoriasis or a history of psoriasis (patient or relative).

b. Arthritis in an HLA-B27-positive male after 6 years old.

c. Ankylosing spondylitis, enthesitis-related arthritis, sacroiliitis with inflammatory bowel disease, Reiter’s syndrome, or acute anterior uveitis, or a history of one of these disorders in a relative.

d. The presence of IgM rheumatoid factor on at least two occasions at least 3 months apart.

2. Oligoarthritis—defined as arthritis affecting one to four joints during the first 6 months of disease. A joint described as “arthritis” is characterized by swelling or limitation in the range of joint movement with joint pain or tenderness, which is for a duration longer than 6 weeks. This should have been observed by a physician that could not identify it as accompanying any other identifiable causes. Two subcategories are recognized: (1) persistent oligoarthritis: affecting a maximum of 4 joints; (2) extended oligoarthritis: affecting a total of more than four joints.

3. Polyarthritis (rheumatoid factor negative)—arthritis affecting five or more joints during the first 6 months; a negative test for RF is needed in order to assess the diagnosis.

4. Polyarthritis (rheumatoid factor positive)—defined as arthritis affecting five or more joints during the first 6 months of disease and two or more positive tests for RF at least with a 3 months difference between the tests results in the first 6 months.

5. Psoriatic arthritis—characterized by arthritis and psoriasis, or arthritis and at least two of the following: dactylitis, nail pitting, psoriasis in a first-degree relative.

6. Enthesitis-related arthritis—arthritis and enthesitis, or either one of the two conditions with at least two of the following: (1) a history of sacroiliac joint tenderness and/or inflammatory lumbosacral pain; (2) the presence of HLA-B27 antigen; (3) onset of arthritis in a male over 6 years of age; (4) acute (symptomatic) anterior uveitis and (5) history of ankylosing spondylitis, enthesitis-related arthritis, sacroiliitis with inflammatory bowel disease, Reiter’s syndrome or acute anterior uveitis in a first-degree relative.

7. Undifferentiated arthritis—defined as arthritis that does not fulfill the criteria of any category or can be included in two or more of the above categories.

## 3. Difficulties in the Classification Criteria—Studies Analysis

JIA’s current ILAR classification criteria provide a unique, globally accepted classification scheme even though changes and clarifications to it are still to be made. In the future, data will be collected for a process that aims to develop a new classification of JIA based on clinical and laboratory evidence, will seek to define disorders characterized by chronic arthritis and analyzed the proposed criteria. For the forms of arthritis classified as others or as idiopathic, it is intended to find homogeneous criteria that would allow it to be included in one of the forms of JIA. Regarding systemic arthritis, which represents 10–15% of JIA cases, there was a marked activation of the immune system played by interleukin-1 and interleukin-6, which is why this disease is considered to be polygenically autoinflammatory [5,6].

In JIA, the pathophysiology involves the activation of the innate immune system. The heterogeneity of the disease is suggested by different sensitivities to interleukin-1 receptor antagonists. It was found that in half of patients, the disease has frequent recurrences followed by remission while the other half have an uninterrupted evolution with persistent arthritis [7,8,9].

Another entity was defined at the JIA level, namely the one with positive ANA antibodies. This entity is characteristic represents up to 50% of JIA cases. It was found that these patients with common characteristics were classified as having RA with negative RF as well as psoriatic arthritis (PsA). It was thought that it would be useful for these forms to be grouped into ANA-positive early onset arthritis, regardless of the number of affected joints and the presence or absence of psoriasis. It was also decided to group as “other JIA conditions” all forms of arthritis that do not meet the criteria of the four diseases mentioned in the classification [10].

The form of arthritis with positive RF represents only 5% of child’s JIA and overlaps with the adult criteria of RA. Only in this form can anti-cyclic citrullinated peptides (CCP) antibodies be considered as important and mandatory diagnostic criteria. RA associated with enthesitis accounts in 5–10% of JIA cases [11]. We sought to identify other objective criteria for homogeneous entities such as symmetry polyarthritis with late-onset negative ANA antibodies, a condition that would be similar to that seen in adults or other forms of SpA such as PsA [12]. ILAR experts also sought evidence that genetic analysis would be important for classifying homogeneous entities. It was decided, however, that clinical and laboratory studies available worldwide should be the basis for new validated criteria to enable a basis for research as well as to be useful in daily clinical practice. They sought to find and use new evidence-based criteria to accurately identify and describe the various diseases included in JIA. These observations are of great importance because in these similar diseases, in children and adults, the same tests can be used and pharmacokinetic and pharmacodynamics data could be used in both groups [13,14,15]. The research is aimed at identifying homogeneous entities as well as distinguishing those changes that are observed or present in both adults and children.

Through the study of the specialized literature, we found that there are possibilities to improve the classification criteria for JIA. In over 30 literature studies, we have found analyses, proposals and controversies related to new criteria for classifying JIA. The most important results are presented below:

There are studies such as Hinze’s that show the difficulties of JIA classification. Patients diagnosed with JIA from three national registries in Germany were analyzed and it was found that a lot of them do not fulfill the ILAR classification criteria, mostly because there were no symptoms of chronic arthritis [16], with the rest of them being difficult to classify or accomplishing the criteria for two entities.

Another study, from Stoll et al., compares early onset-oligoarticular JIA form and early onset-PsA. They analyzed children from two centers, Boston and Texas. The authors concluded that early-onset PsA (2–3 years old) should be removed from the JIA nomenclature of children’s idiopathic arthritis [17].

Petty and colleagues reviewed the JIA’s classification criteria (Durban 1997). The same Petty in 2004 published the revision of the JIA classification, in the second revised edition of the Edmonton classification [18].

Engsw describes the biological basis of juvenile idiopathic heterogeneity (2014) [19].

In 2015, Ombrello and colleagues showed that HLA-DRB and MHC class 2 are a major risk factor for JIA.

In 2017, the same Ombrello showed that genetic architecture can distinguish systemic arthritis and other forms of arthritis with clinical and therapeutic implications [20,21].

JIA is sometimes a real “dilemma” for pediatricians or dermatologists. The lack of updated classification criteria hardens the clinical diagnosis. The insufficient number of pediatricians specialized in rheumatology represents another problem. Our clinical data evaluation comes as a first step for future more extensive studies in the field of JIA.

These observations make us consider that the classification nomenclature should be regarded as a work in progress. Clinicians who are following the evolution of a significant number of children with JIA note that the nomenclature for arthritis is not complete and that many distinct diseases have been put together to be included in studies for a uniform analysis. It was also observed from the analysis of the nomenclature that there are children who do not encompass any category while others have symptoms that would allow them to be included in more than one category [20].

In the case of hard-to-classify children’s diagnoses, we might choose to consider them as having JIA. It is essential to understand that JIA is not a homogeneous entity. The definition of arthritis subtypes should be as relevant as possible for the therapeutic approach, considering that they are based on pathophysiological criteria [21].

In our clinical database, we have found cases that had enthesitis-related JIA associated with the oligoarticular form. What is interesting in the evolution of our cases is that four of them went from the oligoarticular form to the polyarticular form. In these cases, we asked ourselves whether it was a misdiagnosis from the beginning.

## 4. Pediatric Rheumatology International Trials Organization (PRINTO)—A New Classification Based on Four Different Entities

In December 2015, an international consensus conference was convened by researchers of the Scientific Institute for Research, Hospitalization and Healthcare (IRCCS) in order to establish new PRINTO JIA classification criteria. The Pediatric Rheumatology International Trials Organization (PRINTO) found that the classifications that take into account the phenotype do not sufficiently express the specific characteristics of the categories of JIA [1]. Children who develop JIA in the first 5–6 years have a higher risk of uveitis, as well as the phenotypic expression being different within the same JIA category. Studies in population groups have shown that PsA can have two periods of onset. First, it can occur at a young age (2–3 years old) and second, during adolescence. PsA at a young age has similar manifestations to oligoarthritis, and the presence of ANA is higher in this form. Regarding the similarity between non-psoriatic and early-onset-PsA, authors suggest that PsA at an early age is difficult to identify and propose the renunciation of early-onset-PsA from classification [22]. We must approve that the more difficult a classification becomes, the harder it is to be accepted and understood. This drawback could affect therapeutic implementation.

The Pediatric Rheumatology International Trials Organization (PRINTO) also suggests that the actual criteria for the classification of arthritis in children are not sufficient and that there are additional determinant factors in the categories that lead to subclassification. Concerning the division into rigorous disease subtypes, the actual classification considers stratifying the patients who belong to the homogenous studies report groups. In this classification, the term preferred was that of “juvenile idiopathic arthritis”. This classification made by ILAR led to confusion because most clinicians who treat adult patients considered JIA to be the same as RA [23]. There is no correspondence between JIA and adult rheumatoid arthritis. The only similarities are between the form of polyarticular JIA with positive FR and adult rheumatoid arthritis, between arthritis from psoriasis and sacroiliitis. Compared to the systemic form of the adult (Still disease), the systemic form of JIA is very different. Systemic JIA, characterized by an anti-inflammatory phenotype with fever, rash, lymphadenopathy, and marked systemic inflammation. Although considered similar to the AOSD (adult-onset Still disease), these symptoms are found only in the juvenile systemic form of arthritis [24]. Arthritis related to enthesitis or arthritis with enthesitis is an undifferentiated SpA. On the other hand, the forms of polyarthritis with negative RF, as well as undifferentiated SpA, are heterogeneous. These two forms describe patients with early onset but positive to ANA. This manifestation occurs only in childhood and represents the majority of patients with the JIA oligoarticular form [25]. It was also found that the numbers of joints involved, as well as the association with psoriasis, are not important criteria for JIA classification [26]. Therapy has recently advanced with the introduction of biological treatment that aims to inhibit TNF-alpha, IL-1 and IL-6 receptors depending on the subtypes. This biological treatment stopped the evolution of the disease and the osteoarticular changes. From this point of view, a classification based on pathophysiology and genetics is strongly required. For a uniform approach to JIA in children, we should consider the genetic predisposition, the trigger factors and the therapy response [26]. PRINTO concluded that JIA is not a single disease, but a group of different disorders.

After we analyzed data from the medical literature internationally, we identified four entities or four different chronic disorders that fall within the term JIA (Table 2). What is important to note is that three of these disorders are specific to pediatric patients, while one of the forms is similar to the form in adults and is found in a small proportion in children. To simplify the understanding process as well as for the better use of data in research, instead of looking for hypothetical criteria or using the number of joints involved or the presence of psoriasis, it was decided that for now all other forms should be grouped under the term “other JIA”.

### 4.1. Systemic Arthritis

The main modification that is added to the ILAR classification is that patients with fever but without arthritis can now be included. Somehow, this resembles the adult equivalent—adult onset Still disease—but the term “systemic arthritis” was preferred, with a clear emphasis on the systemic symptoms.

Systemic arthritis can be diagnosed based on fever (≥39 °C once a day and returns to ≤37 °C between fever peaks) of unknown origin, daily, for at least 3 consecutive days (excluding infectious, neoplastic, autoimmune, or monogenic autoinflammatory diseases) over a duration of at least 2 weeks and accompanied by two major criteria “or” one major criterion and two minor criteria. The major criteria are: evanescent erythematous rash, arthritis. The minor criteria are: generalized lymph node enlargement associated with hepatomegaly and/or splenomegaly; serositis; arthralgia lasting 2 weeks or longer (in the absence of arthritis); and leukocytosis with neutrophilia.

The proposed criteria for the systemic form of JIA are based primarily on the presence of fever (daily spikes) in line with the ILAR definition. The term *fever reoccurring* has been introduced to underline that fever can go away after at least of 3 days of presentation, reoccurring again over a duration of 2 weeks. In addition to fever one major criterion is necessary. Arthritis is no longer required.

Similarly, the duration of arthritis is not specified because it is very likely in our times a child with a systemic form of JIA that develops fever will most likely be treated with anti-inflammatory medication before 6 weeks of arthritis have elapsed. The consensus of the specialists was to keep the name systemic JIA and to keep systemic JIA among the JIA disorders rather than grouping it with autoinflammatory diseases. This shows the importance of pathophysiological and clinical aspects of the classification. Maybe in the future different subtypes of systemic JIA will help to evaluate and diagnose systemic JIA.

An increased number of complications can characterize the systemic form of JIA. Of these, the most severe and life-threatening is the macrophage activation syndrome (MAS). A study conducted in 2019 analyzed the bronchoalveolar lavage (BAL) fluid from patients with systemic JIA and found out that it contained high levels of IL-18 and the interferon-γ-induced chemokines [27].

### 4.2. RF-Positive Arthritis

Anti-cyclic citrullinated peptide (CCP) antibodies have been added to the definition of the RF (rheumatoid factor)-positive arthritis in addition to the old ILAR classification.

Besides this change, the diagnosis of RF-positive arthritis remains unchanged, being considered the JIA form that resembles rheumatoid arthritis (RA) the most. Arthritis for ≥6 weeks, and two positive tests for RF at least 3 months apart or at least one positive test for antibodies to CCP are the criteria needed for diagnosis.

The number of joints involved is no longer a classification criterion, so the term polyarthritis is no longer used. The duration of arthritis for ≥6 weeks is reported in the criteria. It is considered that the symptoms should ameliorate if arthritis is of another origin such as reactive arthritis, with a self-limiting evolution. Along with the presence of arthritis, the association with at least two positive tests for RF is needed for accuracy of the laboratory results. In addition, the positivity to antibodies to CCP is now considered a valid alternative. Cut-off values for laboratory results will need further studies and clinical trial before they can be introduced in a new classification system.

### 4.3. Enthesitis/Spondylitis

Enthesitis or spondylitis-related JIA is diagnosed based on peripheral arthritis and enthesitis, or arthritis or enthesitis associated with more than 3 months of inflammatory back pain and sacroiliitis on imaging, or rthritis or enthesitis plus two of the following: sacroiliac joint tenderness; inflammatory back pain; HLA-B27 antigen positive; acute (symptomatic) anterior uveitis; and history of a SpA which is present in a first-degree relative. When peripheral arthritis is present, it should persist for at least 6 weeks.

Clinical, genetic and pathophysiologic characteristics are shared between adults with axial spondyloarthritis (SpA) and children with enthesitis-related arthritis (ERA), and between children with ERA and primarily peripheral disease manifestations and adults with peripheral SpA [28]. 

A benefit of this new classification system could be the introduction of therapies approved for adults (certolizumab pegol, ixekizumab, and secukinumab) in children after clinical trials are established. Considering the current lack of effective FDA-approved therapies for ERA, a classification of JIA that introduces enthesitis as a distinctive entity could improve the treatment and general outcome of the pediatric patients [27].

### 4.4. Early Onset ANA+

Early onset ANA-positive disease is an entity characterized by arthritis for ≥6 weeks, and an early age onset (≤6 yrs). Additionally, for a positive diagnosis, two positive ANA tests at least 3 months apart are needed. An important change that was made to the ILAR classification werr the exclusions of systemic JIA, RF-positive arthritis, and enthesitis/spondylitis-related JIA because of the different entities that they constitute.

This condition is a new entry in the JIA classification, and it appears to exist only in children. In Europe and United States of America, this is the most frequent form of JIA and it mostly represents patients that were previously classified in the oligoarticular category. 

A prospective study of the National Pediatric Rheumatological Database (NPRD) in Germany analyzed data from the years 2002 to 2016. Patients with JIA-associated uveitis and with ANA-positive idiopathic anterior uveitis were included in the study. Of the total cohort: 62 ANA-positive patients with idiopathic anterior uveitis constituted the group 1, 688 patients with initial uveitis diagnosis after JIA onset were classified in the group 2, and 61 JIA patients with initial uveitis diagnosis before arthritis onset were included in group 3. It was concluded that ANA-positive idiopathic uveitis and JIA-associated uveitis are not significally different concerning the clinical course of uveitis. This study provided important information on the treatment strategies and the response to corticosteroids and DMARD [29]. Different studies such as this, based on specific symptoms that are measured between patients that fall into different categories are needed in order to assess the relevance of the PRINTO classification.

## 5. Conclusions

The use of the old 2001 Edmonton classification has led to the existence of considerable unclassifiable cases, which had the inclusion criteria for more than one category. In December 2015, an international consensus conference was convened by researchers in order to reach a consensus on new JIA classification criteria. Pediatric Rheumatology International Trials Organization (PRINTO) found that the classifications that take into account the phenotype do not sufficiently express the specific characteristics of the categories of JIA and concluded that JIA consists in a group of different disorders.

After we analyzed the history of the JIA classification system, the data from the medical literature internationally and the new PRINTO classification criteria, we identified four entities or four different chronic disorders that fall within the term JIA and that could be studied extensively in the future. The overall four-entities classification system is an excellent diagnosis tool for pediatricians and pediatric rheumatologists.

However, the transition of the child’s disease subtype from one category to another or the absence of subcategories implies some limitations of the proposed classification system. An overall evaluation of the pathophysiological mechanisms, which will also analyze the etiological mechanisms, will improve the clinical diagnostic. 

## Figures and Tables

**Table 1:** Comparison of ACR ^1^ (1977) and ILAR ^2^ (1997) classification criteria [4].

Classification	ACR ^1^ (1977)	ILAR ^2^ (1997)
Nomenclature	Juvenile rheumatoid arthritis	Juvenile idiopathic arthritis
Minimum duration	≥6 wk	≥6 wk
Age at onset	<16 y	<16 y
≤4 joints in first 6 mo after presentation	Pauciarticular juvenile rheumatoid arthritis	Oligoarticular juvenile idiopathic arthritis: (A) Persistent <4 joints for course of disease; (B) Extended >4 joints after 6 mo
>4 joints in first 6 mo after presentation	Polyarticular juvenile rheumatoid arthritis	1. Polyarticular juvenile idiopathic arthritis-rheumatoid factor negative 2. Polyarticular juvenile arthritis-rheumatoid factor positive
Fever, rash, arthritis	Systemic juvenile rheumatoid arthritis	Systemic juvenile idiopathic arthritis
Other categories included	Exclusion of other forms	1. Psoriatic juvenile idiopathic arthritis 2. Enthesitis-related arthritis 3. Undifferentiated: (A) Fits no other category; (B) Fits more than 1 category
Inclusion of psoriatic arthritis, inflammatory bowel disease, juvenile ankylosing spondylitis	No	Yes

^1^ The American College of Rheumatology; ^2^ International League of Associations of Rheumatology.

**Table 2:** Main characteristics of the four different entities proposed by PRINTO in the new classification system.

Main Characteristics	Systemic Arthritis	RF-Positive Arthritis	Enthesitis/Spondylitis	Early Onset ANA+
**Clinical**	(1) fever; (2) evanescent (nonfixed) erythematous rash; (3) arthritis; (4) generalized lymph node enlargement and/or hepatomegaly and/or splenomegaly; (5) serositis; (6) arthralgia lasting 2 weeks or longer (in the absence of arthritis)	(1) arthritis	(1) arthritis (2) enthesitis (3) sacroiliac joint tenderness; (4) inflammatory back pain; (5) acute (symptomatic) anterior uveitis	(1) arthritis
**Laboratory**	(1) leukocytosis (≥15,000/mm^3^) with neutrophilia.	(1) Rheumatoid factor (RF); (2) Antibodies to cyclic citrullinated peptide (CCP)	(1) sacroiliitis on imaging; (2) HLA-B27 antigen.	(1) ANA tests with a titer ≥1/160 (tested by immunofluorescence)
